# Refractory Anaphylaxis: Data From the European Anaphylaxis Registry

**DOI:** 10.3389/fimmu.2019.02482

**Published:** 2019-10-18

**Authors:** Wojciech Francuzik, Sabine Dölle-Bierke, Macarena Knop, Kathrin Scherer Hofmeier, Ewa Cichocka-Jarosz, Blanca E. García, Roland Lang, Ioana Maris, Jean-Marie Renaudin, Margitta Worm

**Affiliations:** ^1^Department of Dermatology, Venerology and Allergology, Charité—Universitätsmedizin Berlin, Corporate Member of Freie Universität Berlin, Humboldt-Universität zu Berlin, Berlin Institute of Health, Berlin, Germany; ^2^Department of Dermatology and Allergology, Klinikum der Universität München, Munich, Germany; ^3^Department of Dermatology, University Hospital Basel, Basel, Switzerland; ^4^Department of Pediatrics, Jagiellonian University Medical College, Kraków, Poland; ^5^Service of Allergology, Complejo Hospitalario de Navarra, Pamplona, Spain; ^6^Department of Dermatology, Paracelsus Private Medical University Salzburg, Salzburg, Austria; ^7^Department of Paediatrics and Child Health, University College Cork, Cork, Ireland; ^8^Réseau d'Allergo-Vigilance (Allergy Vigilance Network), Vandoeuvre les Nancy, France

**Keywords:** anaphylaxis, adrenaline (epinephrine), beta-blockers, insect venom allergy, drug allergic reactions, vasoconstriction, refractory

## Abstract

Refractory anaphylaxis (unresponsive to treatment with at least two doses of minimum 300 μg adrenaline) is a rare and often fatal hypersensitivity reaction. Comprehensive data on its definition, prevalence, and risk factors are missing. Using the data from the European Anaphylaxis Registry (11,596 cases in total) we identified refractory anaphylaxis cases (*n* = 42) and analyzed these in comparison to a control group of severe anaphylaxis cases (*n* = 4,820). The data show that drugs more frequently elicited refractory anaphylaxis (50% of cases, *p* < 0.0001) compared to other severe anaphylaxis cases (19.7%). Cases elicited by insects (*n* = 8) were more often due to bees than wasps in refractory cases (62.5 vs. 19.4%, *p* = 0.009). The refractory cases occurred mostly in a perioperative setting (45.2 vs. 9.05, *p* < 0.0001). Intramuscular adrenaline (as a first line therapy) was administered in 16.7% of refractory cases, whereas in 83.3% of cases it was applied intravenously (significantly more often than in severe anaphylaxis cases: 12.3%, *p* < 0.0001). Second line treatment options (e.g., vasopression with dopamine, methylene blue, glucagon) were not used at all for the treatment of refractory cases. The mortality rate in refractory anaphylaxis was significantly higher (26.2%) than in severe cases (0.353%, *p* < 0.0001). Refractory anaphylaxis is associated with drug-induced anaphylaxis in particular if allergens are given intravenously. Although physicians frequently use adrenaline in cases of perioperative anaphylaxis, not all patients are responding to treatment. Whether a delay in recognition of anaphylaxis is responsible for the refractory case or whether these cases are due to an overflow with mast cell activating substances—requires further studies. Reasons for the low use of second-line medication (i.e., methylene blue or dopamine) in refractory cases are unknown, but their use might improve the outcome of severe refractory anaphylaxis cases.

## Introduction

Anaphylaxis is a non-homogeneous clinical diagnosis, depending on various triggering and augmenting factors (1). This variability introduces a wide range of possible reaction-symptom severities. Therefore, multiple subtypes of anaphylaxis have been previously identified (i.e., food-dependent exercise-induced anaphylaxis, venom anaphylaxis, biphasic anaphylaxis).

The mainstay of anaphylaxis management is the intramuscular dose of adrenaline (2), but in the most severe cases of anaphylaxis, it might be insufficient to restore a stable patient status. Refractory anaphylaxis (although the established definition is lacking) might be defined as anaphylaxis meeting the criteria by NIAID/FAAN (3) which, after the treatment with at least two doses of minimum 300 μg adrenaline, does not achieve normalization of the clinical symptoms in a given individual. Common elicitors and symptoms of refractory anaphylaxis, as well as the therapeutic strategy for the most severe cases, differ from the usual reactions (4) and call for specific research and targeted guideline development for refractory anaphylaxis cases.

We aimed to distinguish the prevalence of refractory anaphylaxis among anaphylaxis cases and to describe symptoms and factors which may increase the risk of a refractory anaphylaxis episode.

## Methods

**Figure 1:**
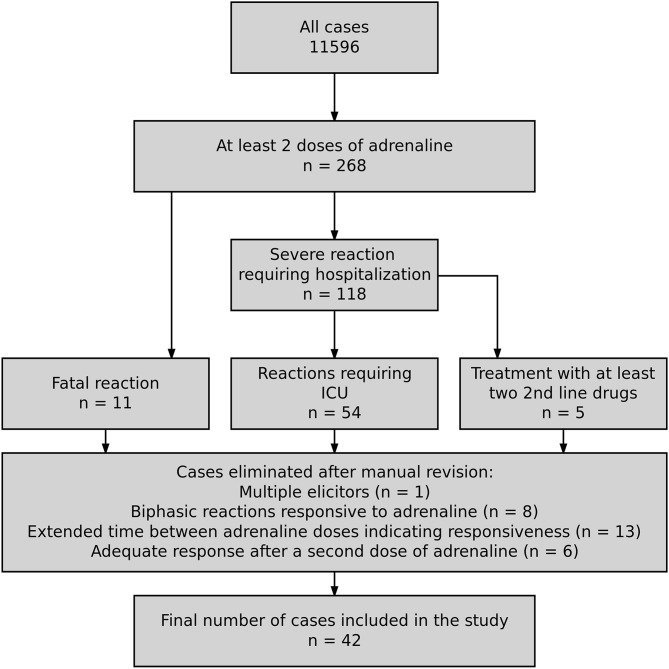
Flowchart illustrating the cases selection process for the final database.

The European Anaphylaxis Registry [described in detail elsewhere (5)] provided data for this analysis (status from March 2018). We selected cases where patients received at least two doses of adrenaline and failed to recover adequately and assigned them to the “refractory anaphylaxis group.” The flowchart in Figure 1 represents the detailed case-selection process.

The final database consisted of 42 cases of refractory anaphylaxis from 7 countries: Germany: 19, Switzerland: 11, France: 6, Austria: 2, Poland: 2, Spain: 1, Ireland: 1. We compared these to a group of severe, non-refractory cases of anaphylaxis. Severe reactions were identified based on the definition by NIAID/FAAN (3) and presented with significant hypoxia, hypotension, confusion, collapse and loss of consciousness, or incontinence. We compared the frequency of various elicitors, symptoms, and factors known to increase the risk of severe anaphylaxis (6) in both groups as well as their management.

We performed a statistical analysis in the R Statistical Package (7). A simple comparison of categorical variables was performed using Fisher's exact test; continuous variables were analyzed using the Mann-Whitney *U*-test. We defined statistical significance as α = 0.05. Data, along with the analysis script, can be accessed at www.github.com/wolass/RefractoryAnaOrg.

## Results

### Refractory Anaphylaxis Accounts for Less Than 0.5% of Severe Anaphylaxis Cases in the Register

The European Anaphylaxis Registry captured 42 cases of refractory anaphylaxis and 4,820 severe, non-refractory anaphylaxis. The frequency of refractory anaphylaxis was 0.37% of all anaphylaxis cases reported in the registry. Each year ~1% (0.853% ± 0.765%) of severe anaphylactic episodes are refractory to treatment with adrenaline. When considering patients who experienced anaphylaxis in a perioperative setting or a medical facility, nearly 3.72% patients present with reactions that do not respond to adrenaline vs. 0.448% in non-medical setting (9.3 times more).

### Increased Frequency of Previous Reactions in Patients With Refractory Anaphylaxis

The mean age at the reaction was 41.4 ± 20.8 years, which did not differ from severe, non-refractory cases, *p* = 0.897). The percentage of males within the refractory anaphylaxis group was 50%. More patients suffered from a concomitant malignant disease in the refractory anaphylaxis group. Most strikingly, patients with refractory reactions more often had a previous anaphylactic reaction in their medical history (*p* = 0.0336). Baseline tryptase levels were significantly higher in the refractory anaphylaxis group as 7 refractory patients (16.7%) had tryptase level above 11.5 μg/L (vs. 7.8%). The demographic summary of refractory cases is shown in Table 1.

**Table 1:** Summary of the refractory anaphylaxis cases.

**Sex**	**Group**	***n***	**Age**	**Cardiologic**	**DM**	**Food allergy**	**Mastocytosis**	**Malignancy**	**Atopic dermatitis**	**Tryptase [median]**
Female	Refractory	22	40.0	31.82	9.09	13.64	9.09	0.00	18.18	5.22
Male	Refractory	20	43.0	30.00	15.00	5.00	5.00	15.00	5.00	7.43
Female	Severe	2421	43.4	20.57	2.27	5.37	2.56	2.19	6.73	4.30
Male	Severe	2399	40.2	22.89	3.58	5.84	2.54	2.08	6.25	4.72
	*p*-value	0.78	0.9	0.19	0.07	1.00	0.10	0.07	0.20	0.01

*Age is represented by a mean value, other variables as fractions [%]. DM, diabetes mellitus, p-value is derived from a Mann-Whitney U-test or a Chi^2^ test for interval and categorical variables, respectively. Refractory group was compared to severe anaphylaxis cases without differentiating into male and female subgroups*.

### Drugs Are the Most Frequent Elicitors of Refractory Anaphylaxis

Refractory anaphylaxis was most commonly elicited by drugs (significantly more often than in severe, non-refractory cases), followed by food and insects (Table 2). The most common drugs eliciting anaphylaxis refractory to adrenaline were antibiotics (19%) and radiocontrast media (RCM, 7.14%). Patients with refractory anaphylaxis more frequently experienced the reaction while undergoing a medical procedure (54.8% vs. 12.3 in severe, non-refractory cases, *p* < 0.0001).

**Table 2:** Summary of elicitors in the refractory anaphylaxis cases and severe, non-refractory anaphylaxis cases as a control.

**Elicitor**	***n***	**%**	**% severe ANA**	**Perioperative [*n*]**	**Food allergy [*n*]**	**Age**	**Male [%]**	***p***
Food	9	21.4	24.1	0	3	17.4	55.6	0.8560
Drugs	21	50.0	19.7	19	0	48.8	42.9	0.0001
Insects	8	19.0	48.1	0	0	46.5	62.5	0.0001
Other	2	4.8	3.0	0	1	38.0	0.0	0.3610
Unknown	2	4.8	5.1	0	0	55.5	50.0	0.0001

*ANA, anaphylaxis, age is represented as a mean, p-value derived from the Fisher's exact test*.

Thirty three percent of food elicited refractory cases had a previously confirmed diagnosis of food allergy. Insect-venom and food allergens most frequently elicited severe cases of anaphylaxis. Refractory anaphylaxis cases were more often elicited by bees than severe, non-refractory cases, *p* = 0.0092 (Table 3).

**Table 3:** Comparison of the specific elicitors from each elicitor-group between the refractory anaphylaxis cases and severe anaphylaxis cases as a control.

**Elicitor**	**Severe ANA [%]**	**Refractory ANA [%]**	***p*-value**
Antibiotics	6.140	19.00	0.0040
X-ray medium	0.954	7.14	0.0080
Muscle relaxant	0.456	4.76	0.0180
Legumes	4.020	7.14	0.2410
Bee venom	9.320	11.90	0.5890
Yellow-jacket venom	33.300	4.76	0.0001

*ANA, anaphylaxis, p-value derived from the Fisher's exact test*.

### Refractory Anaphylaxis Is Life-Threatening

Milder anaphylaxis symptoms (i.e., pruritus, gastrointestinal symptoms, vertigo, chest, and throat tightness) were significantly less present in refractory anaphylaxis cases, whereas respiratory and cardiac arrest, as well as inspiratory and expiratory distress, and death were more often associated with the refractory anaphylaxis cases. Table 4 summarizes the most prominent differences in anaphylaxis symptoms among both groups. Fatal reactions frequently occurred 30–120 min after allergen exposure and were highly associated with refractory cases (26.2% vs. only 0.353% of severe anaphylaxis cases, *p* < 0.0001) In cases where patients responded to life support, but failed to be reanimated due to post-resuscitative complication (e.g., hypoxic brain injury), death occurred in the next 3–8 days.

**Table 4:** Summary of the symptoms in the refractory anaphylaxis cases and severe, non-refractory anaphylaxis cases as a control.

**Symptom**	**Severe ANA [%]**	**Refractory ANA [%]**	***p*-value**
Pruritus	45.40	23.80	0.0050
Skin symptoms	44.80	26.20	0.0190
Respiratory symptoms	62.10	81.00	0.0150
Respiratory arrest	3.03	28.60	0.0001
Chest tightness	8.90	2.38	0.1760
Throat tightness	14.60	7.14	0.2680
Expiratory distress	5.08	26.20	0.0001
Inspiratory stridor	5.31	19.00	0.0020
Loss of consciousness	31.90	40.50	0.2470
Cardiac arrhythmia	3.30	11.90	0.0130
Cardiac arrest	3.07	42.90	0.0001
Vertigo	38.70	14.30	0.0001
Death	0.35	26.20	0.0001

*ANA, anaphylaxis, p-value derived from the Fisher's exact test*.

### Adrenaline i.v. as First-Line Treatment Was Given Frequently in Refractory Anaphylaxis

When evaluating the therapeutic procedures, adrenaline i.v. as a first-line treatment of anaphylaxis was significantly more often given in refractory cases (83.3% vs. 16.7%, *p* < 0.0001). Median time to the second dose of adrenaline was also shorter in refractory cases (2 vs. 15 min in non-refractory cases, *p* < 0.0001).

Corticosteroids i.v. were the second most frequently administered group of drugs in refractory cases (as a first and second-line treatment), outpacing antihistaminic drugs, and volume replacement therapy, and were significantly more often given in refractory cases. Volume replacement therapy was given initially in 61.9% of refractory cases and was sustained only in 19% as the therapy progressed in the hospital environment.

Second-line medication like dopamine, glucagon, and methylene blue were not given in all refractory anaphylaxis cases as well as in severe non-refractory ones. However, patients with refractory anaphylaxis were more frequently admitted to the hospital (85.7%) and treated at the ICUs (78.6%). Table 5 illustrates the therapy of refractory anaphylaxis cases.

**Table 5:** Summary of therapeutic measures in the refractory anaphylaxis cases and severe, non-refractory anaphylaxis cases as a control.

**Therapy**	**Severe ANA [%]**	**Refractory ANA [%]**	***p*-value**
Adrenaline i.m.	8.38	16.70	0.0840
Adrenaline i.v.	12.30	83.30	0.0001
Adrenaline i.v. 2nd line	0.73	40.50	0.0001
Volume	20.50	61.90	0.0001
Volume, 2nd line	3.34	19.00	0.0001
Antihistaminics i.v.	40.90	64.30	0.0030
Antihistaminics i.v. 2nd line	3.84	21.40	0.0001
Corticosteroids, all routes	5.52	7.14	0.5040
Corticosteroids i.v.	48.80	73.80	0.0020
Corticosteroids i.v. 2nd line	5.37	28.60	0.0001
Beta-2-mimetics i.v.	0.66	2.38	0.2500
Beta-2-mimetics inh. 2nd line	0.75	7.14	0.0040
Theophylline i.v.	0.42	0.00	1.0000
100% oxygen	9.42	47.60	0.0001
Dopamine i.v.	0.04	0.00	1.0000
Glucagon i.v.	0.02	0.00	1.0000
Methylene blue	0.00	0.00	1.0000
Hospital admission	28.00	85.70	0.0001
Intensive care	7.55	78.60	0.0001

“*2nd line” refers to the therapy performed by the professional medical emergency team after the initial rescue procedures. ANA, anaphylaxis, p-value derived from the Fisher's exact test*.

### Cofactors of Refractory Anaphylaxis

Patients with refractory anaphylaxis more often had concomitant asthma and malignant diseases in their medical history. Also, other unspecified concomitant conditions were significantly more often reported in refractory cases. Concomitant cardiologic conditions, diabetes, and mastocytosis were similarly frequent in both groups.

Patients with refractory anaphylaxis more often reported concomitant intake of proton pump inhibitors (PPI) and acetylsalicilic acid (ASA) compared with patients with severe non-refractory anaphylaxis. Other (not specified) medications were also more frequent in refractory cases. In 6 cases of refractory anaphylaxis (14.3%) patients reported receiving beta-blockers as a concomitant medication, but none of these patients received a glucagon infusion.

The intensity of physical exercise exceeding the reaction was indifferent between groups, however psychological burden (defined as a stressful event preceding the reaction, rated by the physician) was reported three times more frequently in refractory cases (see Table 6).

**Table 6:** Factors potentially increasing the risk of a severe anaphylaxis investigated in refractory cases.

**Factor**	**Severe ANA [%]**	**Refractory ANA [%]**	***p*-value**
Concomitant asthma	12.10	29.30	0.0030
Concomitant AD	6.82	12.20	0.2000
Concomitant diabetes	5.80	13.20	0.0700
Concomitant cardiologic condition	22.80	31.70	0.1910
Concomitant infection	3.35	4.88	0.6480
History of malignant disease	3.12	10.80	0.0290
Concomitant mastocytosis	2.68	7.32	0.0990
Concomitant other disease—unspecified	15.60	36.60	0.0001
Exercise prior to reaction	28.10	21.40	0.3920
Psychological burden	6.79	26.20	0.0001
Concomitant medication	37.50	60.50	0.0060
ASA	6.13	18.40	0.0080
Beta-blockers	10.40	15.80	0.2810
PPI	5.81	20.60	0.0030
Other drugs	18.20	44.70	0.0001
Alcohol use prior to the reaction	5.51	2.63	0.7210

*ANA, anaphylaxis, p-value derived from the Fisher's exact test*.

## Discussion

### Frequency of Refractory Anaphylaxis and Patients at Risk

Our findings suggest that around 1 in 100 severe anaphylaxis patients will not respond to the standard therapy with adrenaline, commonly outlined in anaphylaxis management guidelines (8). Such cases have the highest risk of a fatal outcome, and therefore need to be treated accordingly. Early use of adrenaline and maybe other second-line drugs (e.g., methylene blue and dopamine) might increase their survival chance.

**Figure 2:**
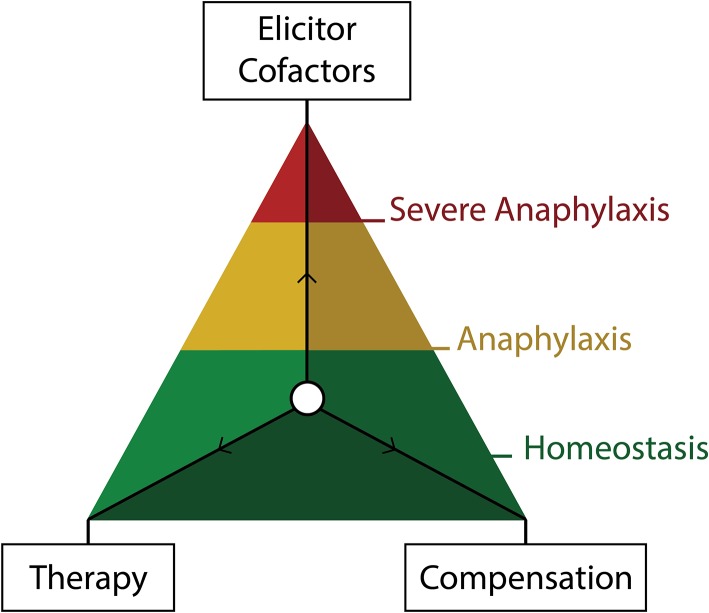
Visual representation of the three forces determining the severity of anaphylaxis. The natural ability of the body to compensate for the anaphylaxis symptoms and therapeutic measures acting to restore homeostasis. Elicitors and cofactors influence the severity of a given episode synergistically.

Overall, the severity of anaphylaxis and its probable transition into a refractory episode might depend on several co-influencing mechanisms: (1) elicitors—the type and dose of an eliciting agent and route of exposure; (2) cofactors—the presence of other individual and external factors which may increase the severity of a given reaction, e.g., concomitant use of a beta-blocker; (3) compensation—how well the affected individual can compensate reaction symptoms, e.g., hypotension; (4) therapy—how fast and accurately the therapeutic interventions were applied (Figure 2).

### Elicitors

Drugs were the most commonly occurring elicitor, which is in concordance with the literature and our previous findings (4). Multiple medications with mast cell activation potential (9) given in a perioperative setting increase the chance of a hypersensitivity reaction and drug interactions (10). A recent study on IgE-independent anaphylaxis showed that perioperative drugs (i.e., rocuronium, tubocurarine, fluoroquinolones, atracurium) might trigger anaphylaxis by activating mast cells directly through the MRGPRX2 receptor (11). Therefore, patients undergoing surgical procedures and having a history of anaphylaxis should remain under extraordinary caution.

Antibiotics, although commonly triggering IgE-dependent reactions (12), are rarely reported in the literature as the cause of refractory anaphylaxis. RCM, on the other hand, commonly elicit IgE-independent hypersensitivity episodes that are refractory to adrenaline and are responsible for 1–5 deaths per 100,000 administrations (13). We previously reported RCM to be the most frequent elicitor of refractory anaphylaxis (4). It might be that RCM promotes complement activation (14) and thus—unspecific activation of multiple immune cell classes (mast cells, basophils, platelets, and neutrophils). Therefore, treatment with adrenaline may be less effective in these reactions.

Yellow-jacket-stings elicited only a few refractory cases. Therefore, the ratio of yellow-jacket to honey-bee venom elicited anaphylaxis was inverted in the refractory group. Reasons for this observation might include higher allergen exposition due to either extended exposition to the allergen (bee's sting remains in the skin) or the venom volume being 10 times higher than in a wasp sting (15).

### Risk Factors

Although we have previously identified higher age and male sex to be associated with severe anaphylaxis (6), these factors seem to be less critical in refractory anaphylaxis cases. By contrast, we previously associated concomitant asthma with less severe anaphylaxis in our registry data (6). However, the present analysis, as well as other previous data (16), suggest concomitant asthma as a risk factor for a severe episode. Probably the control of the asthmatic condition is essential in this context and should be therefore evaluated in patients at risk. Moreover, it might be possible that during a refractory episode the bronchospasm is additionally triggered via mediators derived from basophils and eosinophils or the vegetative nerve system what makes it refractory to adrenaline and results in a prolonged anaphylactic episode. Accordingly, we observed more respiratory distress symptoms in the refractory anaphylaxis cases.

Baseline serum tryptase (BST) levels were increased in patients with refractory anaphylaxis, although only in 7 patients they exceeded the threshold level of 11.5 ng/mL—what is commonly recognized as elevated (17). BST may reflect pro-β tryptase due to increased body mast cell content (18) or result from alpha-tryptasemia due to multiple copies of *TPSAB1* gene (19). Irrespective of the mechanism—increased BST has been shown to correlate with the severity of anaphylaxis (20).

The higher rate of malignancy in patients with refractory anaphylaxis diseases might be a confounder as certain intravenous chemotherapeutics are well known to directly activate mast cells (e.g., taxanes) (21). On the other hand, it is possible that patients with reported malignancies were older (mean age of 34.6 vs. 58.3 years, *p* < 0.001) and therefore less able to compensate the anaphylactic shock.

Psychological stress in temporal proximity to the reaction might increase the severity of the reaction as we previously reported (6). Stress induces the complement cascade activation (22). Substance P, released during stress (23), is a known vasodilator (24) and can activate mast cells directly (25). These mediators may contribute to an increased mast cell response and more severe anaphylaxis. Some published case reports indicated stressful event as the anaphylaxis trigger (26).

Wölbling et al. indicated the role of PPI as a risk factor for severe anaphylaxis by prolonging the exposition to an oral allergen (preventing its degradation due to lower gastric acid production) (27). Moreover, there are reports on PPI themselves as elicitors of anaphylaxis (28). We observed a significantly higher rate of PPI intake in the refractory anaphylaxis group. Its mechanistic contribution to the increase in severity of an episode or being responsible for its refractoriness cannot be estimated based on this observation.

Concomitant ASA use was also associated with refractory cases more frequently than in severe anaphylaxis. The role of ASA on increasing the severity of anaphylaxis has previously been implicated by increasing the intestinal absorption of allergens (27). Nevertheless, as ASA might be both a cofactor and elicitor of anaphylaxis (29) and is highly correlated with another cofactor—concomitant cardiologic conditions—it is tough to evaluate its isolated influence on the anaphylaxis severity.

### Symptom Compensation

Age is the most critical factor influencing the risk of developing severe anaphylaxis (6). We and others have shown that older age may correlate with the decreased ability to retain homeostasis on increased strain (30). Patients who underwent refractory anaphylaxis more often had perioperative reactions and therefore, a decreased ability to compensate the reaction symptoms with reflexive renal or cardiopulmonary response (31).

Compensation mechanisms demonstrated in animal models indicated that anaphylactic hypotension activates the hypothalamic paraventricular nucleus, medullary nucleus tractus solitarii, and rostral ventrolateral medulla, independently of the baroreflex pathway. Further, it stimulates efferent sympathetic nerve activity to the adrenal gland and kidney to restore blood pressure (32).

### Therapy

Physicians and surgeons often used adrenaline i.v. as first-line therapy in refractory cases, probably because most of them occurred in a medical setting. However, other second-line therapeutic options were rarely used. Grabenhenrich et al. (2) recently evaluated the epinephrine usage in anaphylaxis patients and concluded that, even in this state of the art drug, significant discrepancies between recommended use and actual treatment practice exist. Similarly, US studies documented poor adherence in patients and caregivers to anaphylaxis guidelines recommending more than one adrenaline autoinjector available at all times (33). Therefore, more effort needs to be dedicated to promote and develop consensus guidelines as practically as possible in order to increase adherence.

Methylene blue and vasopressors have been described to successfully restore refractory hypotension and recommended by management guidelines (8), but their actual use in anaphylaxis patients is scarce. There are multiple reports of successful methylene blue use in refractory anaphylaxis (4). Evora (34) reported 6 cases of refractory, perioperative anaphylaxis (to RCM and penicillin) which responded to methylene blue i.v. within minutes. Methylene blue blocks the guanylate cyclase and therefore prevents further nitric-oxide-dependent vasodilation in a distributive shock (35). Its potential role in neuroprotection has also been indicated (36).

Surprisingly, even though multiple anaphylaxis management guidelines recommend glucagon infusions in cases of concomitant beta-blocker therapy in anaphylactic patients (8, 37, 38), it has not been administered in any of the severe or refractory cases. Similarly, Royal Collage of Anesthetists reported one glucagon administration in 266 severe intraoperative anaphylaxis episodes (39). Glucagon has been reported to successfully relive refractory anaphylaxis (40, 41) by directly activating the adenylyl cyclase and therefore bypassing the β-adrenergic receptor blockade (40).

### Limitations and Strengths

The low number of refractory cases prevented us from analyzing the data with more advanced statistical models. However, our analysis is the first report on a patient cohort exceeding 30 refractory anaphylaxis cases.

The definition of refractory anaphylaxis is not universal, and we had to assume it based on the answers to our online questionnaire. If a fatal reaction occurred before the second dose of adrenaline was administered to the patient (although, it might have been refractory)—it was not categorized as refractory as we defined at least two doses of minimum 300 μg adrenaline for refractory anaphylaxis.

The therapy with adrenaline was not weight-adapted, and patients received multiple fixed doses of adrenaline ranging from 300 to 1,000 μg. It is possible that patients experiencing anaphylaxis refractory to standard doses of adrenaline were treated with subtherapeutic doses in the initial phase of the reaction due to their obesity. However, we did not gather data on the patient's weight, and therefore, we cannot conclude how it influenced the therapy-outcomes with fixed doses of adrenaline. Nevertheless, repeated doses of adrenaline should be sufficient to alleviate the symptoms in such patients if the weight would be the only reason for the refractoriness of anaphylaxis.

The comparison of refractory anaphylaxis with patients suffering from severe anaphylaxis enabled us to distinguish patients with a higher risk of developing a refractory episode. However, we cannot address the question which patients had a higher risk of experiencing anaphylaxis per se.

## Conclusion

Refractory anaphylaxis is a rare form of a life-threatening hypersensitivity reaction with high mortality. Its elicitors and cofactors differ from other anaphylaxis cases, and the management of refractory anaphylaxis needs to improve. Although, more studies need to be conducted to understand better the pathomechanisms involved in refractory anaphylaxis, we propose to increase the use of second-line medication such as methylene blue, vasopressin and (in suspicion of a beta-adrenergic blockade) glucagon in cases where 2 doses of adrenaline did not result in a rapid normalization of anaphylaxis symptoms.

## Data Availability Statement

All datasets generated for this study are included in the manuscript and/or the supplementary files.

## Ethics Statement

The ethics commission and data protection officer of the Berlin Charité Hospital approved the study (EA1/079/06). Patients gave their informed consent for the inclusion of their medical information in the registry.

## Author Contributions

WF wrote the original manuscript and performed the statistical analysis. SD-B consolidated the data and revised the manuscript critically. MK, KS, EC-J, BG, RL, IM, J-MR, and MW provided data, revised the manuscript critically, and accepted the final version.

### Conflict of Interest

The authors declare that the research was conducted in the absence of any commercial or financial relationships that could be construed as a potential conflict of interest.
